# Novel Postzygotic Variants Associated With Hypomelanosis of Ito Expand the ACTB‐Related Neurocutaneous Disease Spectrum

**DOI:** 10.1111/cge.70188

**Published:** 2026-05-28

**Authors:** Estella Castillon, Paul Rollier, Didier Bessis, Laurent Pasquier, Caroline Racine, Alexis Praga, Pierre Vabres, Bertille Bonniaud, Paul Kuentz, Laurence Faivre

**Affiliations:** ^1^ Université Bourgogne Europe, Inserm, CTM UMR1231, Equipe GAD, FHU TRANSLAD, Centre de Génétique, Centre de Référence Anomalies du Développement et Syndromes Malformatifs, Centre de Référence Déficiences Intellectuelles de Causes Rares Dijon France; ^2^ Univ Rennes, CHU Rennes (Centre de Référence Anomalies du Développement et Syndromes Malformatifs), CNRS, IGDR‐UMR6290 Rennes France; ^3^ Service de Dermatologie, Centre de Référence Des Maladies Rares de la Peau et Des Muqueuses D'origine Génétique, CHU Montpellier Montpellier France; ^4^ Univ Rennes, CHU Rennes (Centre de référence Déficiences Intellectuelles de Causes Rares), CNRS, ARENES‐UMR 6051 Rennes France; ^5^ Université Marie et Louis Pasteur, CHU Besançon Franche‐Comté, Oncobiologie Génétique Bioinformatique, FHU TRANSLAD Besançon France; ^6^ Université Bourgogne Europe, CHU Dijon Bourgogne, Centre de Référence Des Maladies Rares de la Peau et Des Muqueuses D'origine Génétique MAGEC Dijon France; ^7^ Université Bourgogne Europe, Inserm, CTM UMR1231, Equipe GAD Dijon France

**Keywords:** ACTB, Blaschko's lines, deep next‐generation sequencing, hypomelanosis of Ito, hypopigmentation, neurocutaneous phenotype, pigmentary mosaicism

## Abstract

Several clinical entities are associated with *ACTB* pathogenic variants. Most notably, constitutional missense gain‐of‐function variants are linked to Baraitser‐Winter cerebrofrontofacial syndrome, and recurrent somatic gain‐of‐function Arg147 variants are reported in Becker's nevus or in smooth muscle hamartomas. We describe three individuals with mosaic hypopigmentation following Blaschko's lines, associated or not with neurodevelopmental features, with postzygotic *ACTB* variants identified with deep next‐generation sequencing on skin biopsy from an affected area. We identified the same missense p.(Arg335His) variant in individuals #1 and #3, already reported in a constitutional state in a fetus. Individual #2 carried an unreported in‐frame insertion–deletion (p.(Ser348_Leu349insPheHisLeuProProSerIle)). Thus, we describe a previously unreported phenotype related to postzygotic *ACTB* variants with hypopigmentation associated or not with neurodevelopmental features, distinct from Becker presentations, bridging constitutional neurodevelopmental and somatic cutaneous phenotypes.

## Graphical abstract

We describe a previously unreported phenotype related to postzygotic ACTB variants with hypomelanosis of Ito, characterized by hypopigmentation associated or not with neurodevelopmental features, distinct from Becker presentations, bridging constitutional neurodevelopmental and somatic cutaneous phenotypes.

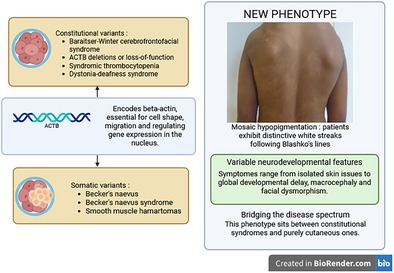

## 1 Introduction

*ACTB* is located on the short arm of chromosome 7, encoding beta‐actin. Molecules from the actin family are components of the actin cytoskeleton and play a central role in the cytoplasm, with several housekeeping functions such as regulation of cell shape and migration. Beta‐actin is ubiquitous and expressed in the nucleus as well, where it regulates gene expression, cell division, and proliferation [1, 2].

Multiple clinical entities have been described in association with *ACTB* pathogenic variants. Baraitser‐Winter cerebrofrontofacial syndrome (BRWS) (MIM#243310) is an autosomal dominant disorder usually caused by constitutional missense de novo gain‐of‐function pathogenic variants in *ACTB* or *ACTG1* (encoding the paralog gamma‐actin) [2, 3]. It is a rare developmental disorder with recognizable craniofacial features (prominent metopic ridge, highly arched eyebrows with widely spaced eyes, bilateral ptosis, bulbous nose with broad tip, long and smooth philtrum) and developmental delay (DD)/intellectual disability (ID) in almost all patients. Other features are frequent but inconstant, including pachygyria, microcephaly, seizures (correlated to structural brain anomalies), genitourinary abnormalities, gastrointestinal dysfunction, congenital heart defects, hearing loss, iris or retinal coloboma and neuromuscular abnormalities [3].

*ACTB* deletions and constitutional loss‐of‐function variants cause another malformative syndrome, with inconstant and milder DD/ID than in BRWS. Features include morphological brain abnormalities, congenital cardiac and renal anomalies, inconstant growth delay, and facial features distinct from BRWS [2, 4]. Other specific constitutional loss‐of‐function variants, clustering in exons 5 and 6 and expected to escape nonsense RNA mediated decay, are associated with syndromic thrombocytopenia (MIM#620475) [1].

Moreover, the particular missense c.547C>T; p.(Arg183Trp) variant is associated with dystonia‐deafness syndrome (MIM#607371), with drug‐resistant dystonia, early‐onset sensorineural deafness, variable morphologic features, DD/ID, scoliosis and epilepsy [3, 4, 5, 6]. Molecular studies have shown that this variant has a gain‐of‐function effect [7].

*ACTB* somatic gain‐of‐function variants have been described in several cutaneous phenotypes, including the recurrent p.(Arg147Ser/Cys) variants in Becker's nevus (MIM#604919) and smooth muscle hamartomas (MIM#620470). Becker's nevus is a type of cutaneous hamartoma, with circumscribed hyperpigmentation and hypertrichosis [8]. In rare cases, it is associated with musculoskeletal anomalies, unilateral breast hypoplasia, cardiomyopathy and developmental delay, termed Becker's nevus syndrome [8]. The same p.(Arg147Ser/Cys) somatic variants and others (c.411G>T; p.(Gln137His) and c.331A>G; p.(Asn111Asp)) were identified in patients presenting with smooth muscle hamartomas [9].

Here, we describe a new clinical entity, observed in three individuals, with molecular and clinical features distinct from the phenotypes previously described in association with *ACTB*. They present with mosaic hypopigmentation following Blaschko's lines, associated or not with neurodevelopmental disorder (NDD).

## 2 Material and Methods

All three individuals were identified during routine clinical care in the same reference laboratory through sequencing of a gene panel targeting mosaic skin disorders. Deep next‐generation sequencing (NGS) was performed on DNA extracted from skin biopsies from the affected area and blood samples for all individuals and from saliva for individual #2.

Targeted sequencing was performed using a custom 55‐gene panel (SOPHiA Genetics (Lausanne, Switzerland)) covering the coding exons and flanking intronic regions of *ACTB* (NM_001101.5). Libraries were prepared by targeted capture and sequenced using paired‐end reads (~300‐bp) on a MiSeq platform (Illumina (San Diego, CA, USA)). Reads were aligned to the GRCh38 reference genome, with a mean coverage depth of ~2500×.

Clinical and molecular data were collected for all three individuals.

Written consent was obtained for all three individuals.

## 3 Results

We identified the c.1004G>A; p.(Arg335His) variant in Individuals #1 and #3, with a variant allele fraction (VAF) of 13.8% (292/2114 reads) and 25.1% (795/3168 reads), respectively. In Individual #2, we identified an unreported in‐frame insertion/deletion c.1044delinsCTTCCACCTTCCACCTTCCATC; p.(Ser348_Leu349insPheHisLeuProProSerIle) in 26.6% of reads (451/1695). A saliva sample showed the variant in 83/3786 reads (2.2%), thus confirming its mosaic nature. Blood was negative for all three individuals.

The p.(Arg335His) variant identified in individuals #1 and #3 has already been reported once in a heterozygous fetus with large cystic hygroma and omphalocele, cystic kidneys and small bladder [10]. To the best of our knowledge, the p.(Ser348_Leu349insPheHisLeuProProSerIle) variant has never been reported.

**FIGURE 1:**
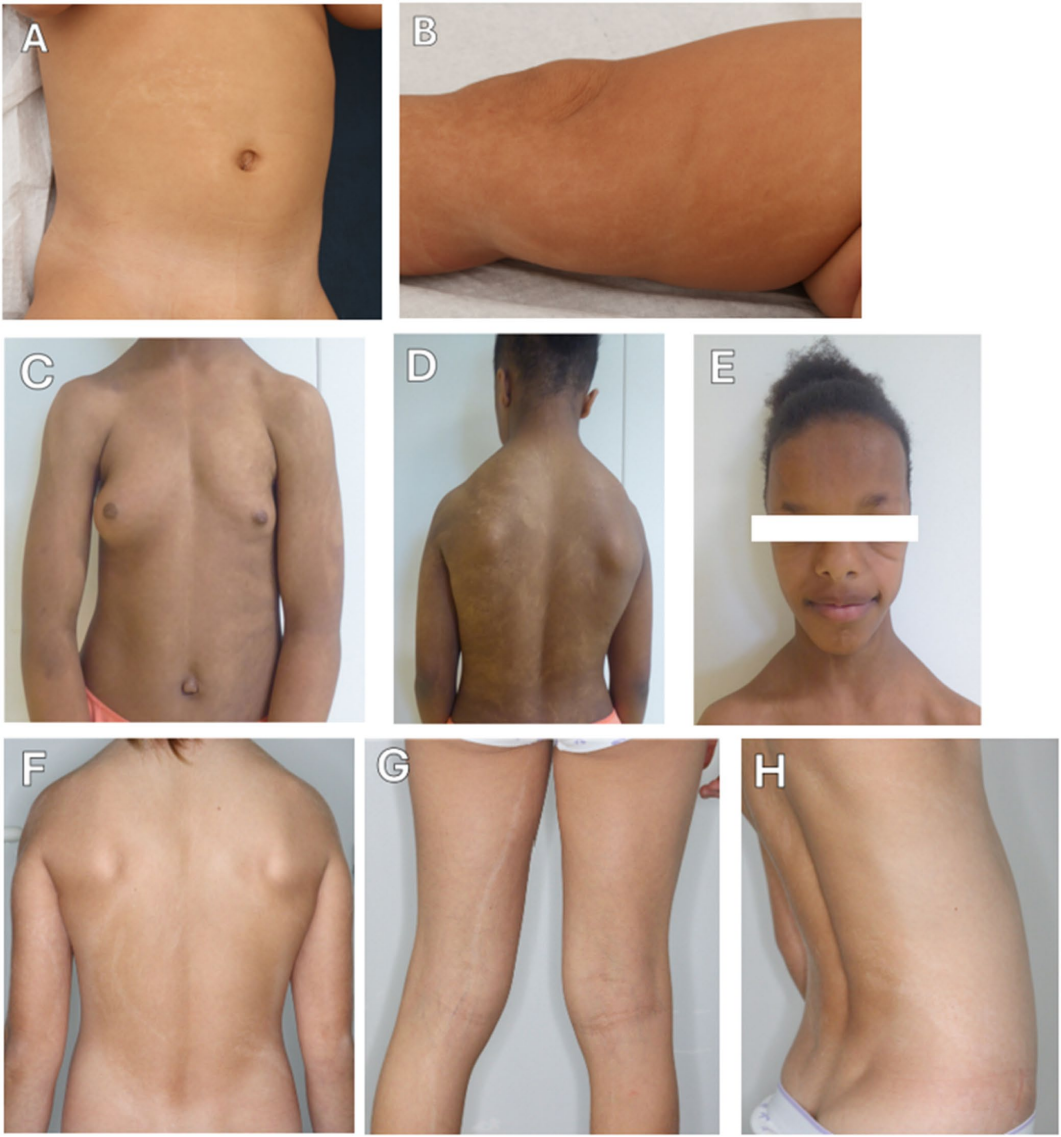
(A and B) Individual #1 at 18 months. (C–E) Individual #2 at 9 years. (F–H) Individual #3 at 9 years.

Individual #1 (Table S1) was a 5‐year‐old boy, born from nonconsanguineous parents, and referred for developmental delay and hypopigmented skin lesions. An adult relative on his mother's side presented with NDD. There was no familial history of skin lesions. From 1 month old, his parents noticed hypopigmented streaks on the right side of his body (inner thigh, torso and armpit), following Blaschko's lines, with a “splashing” pattern (Figure 1). He also presented with macrocephaly (+2.9 SD at 5 years), without notable morphological features. He had astigmatism, hypermetropia and strabismus. He had no dental history. Moderate axial hypotonia was noted during his childhood. He held his head at 8 months, sat at 14 months, crawled at 18 months, and started walking between 2 and 2.5 years of age. He presented with speech development delay and global learning delay. He benefited from speech therapy, physical therapy and psychomotor therapy and a dedicated school aide. At 17 months, brain MRI was normal.

Individual #2 (Table S1) was a 10‐year‐old girl, born in Eritrea from nonconsanguineous parents, and referred for developmental delay and hypopigmented skin lesions. Her siblings, including her twin brother, were free from medical concern. Since birth, she had linear or whorled streaks of hypopigmentation following Blaschko's lines, on the torso, abdomen and four limbs, as well as facial features including hypertelorism, broad tip and base of the nose, malar hypoplasia, sparse eyebrows and small, round ears (Figure 1). She also had ectopic teeth, a small and narrow jaw, narrow palate, and dental malocclusion, requiring orthodontic care. She had asymmetric development of breasts. Cardiac ultrasound showed moderate tricuspid regurgitation, with a morphologically normal valve. She walked around 2 years of age (later than her twin) but did not present speech delay. She benefited from speech therapy for pronunciation difficulties, but there was no formal diagnosis of language delay or impairment. Brain MRI was normal.

Individual #3 (Table S1) was a 9‐year‐old girl, referred by dermatology. Her personal and familial history was unremarkable. She presented with isolated hypopigmentation on the limbs and torso (Figure 1). She attended mainstream school. Given her molecular results, a brain MRI and cardiac ultrasound were performed at 10 years old and were unremarkable.

## 4 Discussion

Here, we report three individuals with postzygotic variants in *ACTB* distinct from the recurrent p.(Arg147Ser/Cys) variants. Beta‐actin plays a crucial role in the cytoplasmic cytoskeleton as well as in the nucleus, where it regulates gene expression. Several clinical entities have been associated with *ACTB* pathogenic variants. We describe here a previously unreported mosaic neurocutaneous phenotype, including hypopigmentation along Blaschko's lines.

Hypopigmentation following Blaschko's lines has been described in the context of neurocutaneous disorders, where the skin condition is associated with a variety of multisystem abnormalities, notably involving the nervous system [11]. Such pigmentary mosaicism can occur in the context of nonrecurrent mosaic chromosomal anomalies [12], but monogenic postzygotic variants have also been identified. Alterations of cytoskeletal dynamics are expected to impair melanogenesis, as melanosomes rely on an intact cytoskeletal network for their transfer to keratinocytes [13]. Genes that have been shown to be involved are part of cell growth, cytoskeletal dynamics, and cell cycle control, such as *MTOR* [14], *RHOA* [15], or X‐linked *TFE3*, *USP9X*, *PHF6*, and *DDX3X* [16].

One plausible hypothesis for the distinct pigmentary phenotypes associated with *ACTB* variants involves timing‐ and tissue‐specific effects of the variant during embryonic development. Early postzygotic events could affect melanocyte migration or survival along Blaschko's lines, potentially leading to hypopigmentation, whereas later, more localized somatic events may lead to clonal melanocyte expansion or altered melanocyte–keratinocyte interactions, which could account for hyperpigmented lesions. Additionally, variant‐specific effects on actin cytoskeleton dynamics may differentially impact melanocyte function, including melanosome transport, cell adhesion, and proliferation, thereby modulating pigmentation outcomes.

In addition to developmental timing, hypopigmentation in mosaic *ACTB* disorders may involve cellular interference between wild‐type and mutant populations, similar to the mechanism described in *PCDH19*‐related epilepsy [17]. A dominant‐negative effect within the actin cytoskeleton could disrupt melanocyte function or survival along Blaschko's lines. Alternatively, a transdominant‐negative mechanism—analogous to collagenopathies—might occur if mutant beta‐actin impairs the dynactin complex. This would hinder dynein‐mediated transport, leading to a more severe phenotype than loss‐of‐function alone through disrupted intracellular trafficking and cytoskeletal dynamics at the tissue level [18].

In conclusion, postzygotic *ACTB* variants are associated with a novel neurocutaneous phenotype. These findings support a mechanistic continuum between constitutional and mosaic disease and highlight *ACTB* as a key diagnostic target for neurocutaneous mosaic disorders, illustrating how cytoskeletal gene‐related phenotypes depend on spatiotemporal variant distribution.

## Author Contributions

E.C. wrote the manuscript. L.F. and P.K. supervised the study. P.R., D.B., L.P., L.F., C.R. and E.C. collected patient clinical data. P.K. and A.P. performed genetic sequencing. P.V. and B.B. provided dermatological expertise and critical review. All authors approved the final manuscript.

## Funding

This work was supported by grants from the European Union through the FEDER programs.

## Conflicts of Interest

The authors declare no conflicts of interest.

## Supporting information


**Table S1:** Clinical, molecular and imaging features of the three reported individuals.

## Data Availability

The data that support the findings of this study are available from the corresponding author upon reasonable request.
