# Supplementary material for: Novel Postzygotic Variants Associated With Hypomelanosis of Ito Expand the ACTB‐Related Neurocutaneous Disease Spectrum
Source: Clin Genet. 2026 May 28;110(3):369–73. doi: 10.1111/cge.70188 (PMC13431713; doi:10.1111/cge.70188)
Supplement: Supplementary file 1 — Table S1: Clinical, molecular and imaging features of the three reported individuals. [file CGE-110-369-s001.docx]

Supplementary - Table 1 : Clinical, molecular and imaging features of the 3 reported individuals

| Features | Individual #1 | Individual #2 | Individual #3 |
| --- | --- | --- | --- |
| Age (years) | 7 | 11 | 10 |
| Sex | M | F | F |
| *ACTB* variant | NM_001101.5:c.1004G>A; p.(Arg335His) | NM_001101.5:c.1044delinsCTTCCACCTTCCACCTTCCATC; p.(Ser348_Leu349insPheHisLeuProProSerIle) | NM_001101.5:c.1004G>A; p.(Arg335His) |
| Cutaneous features | Hypopigmented streaks on right thigh, torso and armpit, following Blaschko lines | Linear or whorled streaks of hypopigmentation following Blaschko’s lines, on the torso, abdomen and four limbs | Hypopigmented streaks on all limbs and torso. |
| Development | Global developmental delay, learning difficulties. | No speech delay, but required speech therapy. Mild motor delay. | No developmental delay. |
| Brain MRI | Normal at 17 months-old | Normal | Normal at 10 years-old |
| Facial features | No notable morphological feature | Hypertelorism, broad nasal tip and base of the nose, sparse eyebrows and small, round ears | No notable morphological feature |
| Other features | Macrocephaly, astigmatism, hypermetropia, strabismus | Ectopic teeth, small jaw, narrow palate, dental malocclusion, moderate tricuspid regurgitation  Asymmetric development of breasts | None |
